# Genome-Based Identification of the Dof Gene Family in Three *Cymbidium* Species and Their Responses to Heat Stress in *Cymbidium goeringii*

**DOI:** 10.3390/ijms25147662

**Published:** 2024-07-12

**Authors:** Xin He, Meng-Meng Zhang, Ye Huang, Jiali Yu, Xuewei Zhao, Qinyao Zheng, Zhong-Jian Liu, Siren Lan

**Affiliations:** 1 College of Forestry, Fujian Agriculture and Forestry University, Fuzhou 350002, China; 5220422102@fafu.edu.cn (X.H.); 1220428020@fafu.edu.cn (M.-M.Z.); llzy6860@163.com (J.Y.); zxw6681@163.com (X.Z.); 2 Key Laboratory of National Forestry and Grassland Administration for Orchid Conservation and Utilization, College of Landscape Architecture and Art, Fujian Agriculture and Forestry University, Fuzhou 350002, China; 5221726097@fafu.edu.cn (Y.H.); qinyaozheng@fafu.edu.cn (Q.Z.)

**Keywords:** Dof genes, *Cymbidium*, expression analysis, heat stress

## Abstract

As an important genus in Orchidaceae, *Cymbidium* has rich ecological diversity and significant economic value. DNA binding with one zinc finger (Dof) proteins are pivotal plant-specific transcription factors that play crucial roles in the growth, development, and stress response of plants. Although the Dof genes have been identified and functionally analyzed in numerous plants, exploration in Orchidaceae remains limited. We conducted a thorough analysis of the Dof gene family in *Cymbidium goeringii*, *C. ensifolium*, and *C. sinensis*. In total, 91 Dof genes (27 *CgDof*s, 34 *CeDof*s, 30 *CsDof*s) were identified, and Dof genes were divided into five groups (I–V) based on phylogenetic analysis. All Dof proteins have motif 1 and motif 2 conserved domains and over half of the genes contained introns. Chromosomal localization and collinearity analysis of Dof genes revealed their evolutionary relationships and potential gene duplication events. Analysis of *cis*-elements in *CgDof*s, *CeDof*s, and *CsDof*s promoters showed that light-responsive *cis*-elements were the most common, followed by hormone-responsive elements, plant growth-related elements, and abiotic stress response elements. Dof proteins in three *Cymbidium* species primarily exhibit a random coil structure, while homology modeling exhibited significant similarity. In addition, RT-qPCR analysis showed that the expression levels of nine *CgDofs* changed greatly under heat stress. *CgDof03*, *CgDof22*, *CgDof27*, *CgDof08*, and *CgDof23* showed varying degrees of upregulation. Most upregulated genes under heat stress belong to group I, indicating that the Dof genes in group I have great potential for high-temperature resistance. In conclusion, our study systematically demonstrated the molecular characteristics of Dof genes in different *Cymbidium* species, preliminarily revealed the patterns of heat stress, and provided a reference for further exploration of stress breeding in orchids.

## 1. Introduction

Plants have various strategies to regulate gene expression, among which transcription factors (TFs) play a vital role in regulating genes at the transcriptional level [1,2]. DNA binding with one zinc finger (Dof) proteins are one of the identified plant-specific TF families. The Dof proteins belong to C2H2-type zinc finger family proteins and have multiple roles such as stress response, phytohormones, photosynthesis, flower induction, seed germination, and light regulation [2,3,4,5]. Compared to the N-terminus, the C-terminal region of Dof proteins exhibits a high degree of variability. Dof transcription factors generally contain 200 to 400 amino acids, and the highly conserved Dof domain is usually located at the N-terminus, which has been identified as a DNA-binding domain and includes approximately 52 amino acids with a C2C2 zinc finger structure [3,6,7]. The Dof domain was proven to function as a Cys2/Cys2 zinc finger domain, which possesses a longer putative loop than that found in zinc finger domains of GATA1 and steroid hormone receptors [5]. The Dof domain is a multifunctional domain, not only involved in binding to DNA but also crucial for multiple protein–protein interactions, thus being regarded as a bi-functional domain that combines with DNA and interacts with other proteins [3,8,9,10].

Since the isolation of the first Dof gene, *ZmDof1*, from maize (*Zea mays*), homologs have been found in a variety of plants [11,12,13]. Dof genes have been shown to be highly expanded in terrestrial plants and increasingly identified in plants such as *Dendrobium huoshanense* (22), *Selenicereus undatus* (26), *Oryza sativa* (30), *Arabidopsis thaliana* (36), *Solanum tuberosum* (36), *Populus trichocarpa* (41), and *Brassica rapa* (76) [3,13,14,15,16,17,18,19]. Dof genes have been found in many plants, and their functions have aroused wide interest. First, Dof genes are involved in a range of crucial plant growth and developmental processes. Studies have shown that several Dof genes of *Vitis vinifera* perform their functional roles mainly during flower, berry, and seed development, highlighting their importance for *V. vinifera* growth and production [20]. Cai et al. found that the expression patterns of *PsDof* genes are consistently upregulated in various tissues and under different stress conditions, suggesting their pivotal involvement in both plant development and stress response [16]. Additionally, Dof proteins are also integral components in both biotic and abiotic stress responses. Transcription factor *ZmDof22* enhances drought tolerance by regulating stomatal movement and antioxidant enzyme activities in *Z. mays* [21]. Jin et al. identified five Cycling DOF Factors (CDFs) in potato, including *StCDF1*/*StDof19*, *StCDF2*/*StDof4*, *StCDF3*/*StDof11*, *StCDF4*/*StDof24*, and *StCDF5*/*StDof15*, which are homologs of *Arabidopsis* CDFs and may serve as the initial step in the abiotic stress response signaling cascade [19]. Furthermore, Chen et al. analyzed the induced expression of *PeDofs* under high-temperature stress during three periods, in which *PeDof-11* was significantly induced with high expression [22]. Although Dof gene families in many plants have been extensively studied, the characteristics and roles of Dof genes in orchids have not been fully studied.

*Cymbidium* (Orchidaceae) has about 50 species and was divided into three subgenera (*Cymbidium*, *Cyperorchis*, and *Jensoa*). In addition, it is one of the most popular orchids in horticulture due to its high ornamental value [23]. Li et al. studied the response mechanisms of two different *Cymbidiums* (*Cymbidium tracyanum* and *C. sinense*) to drought stress and found that *C. sinense* (remedy strategy) and *C. tracyanum* (precaution strategy) adopt different adaptive strategies when facing drought stress [24]. The emergence of sequencing technologies has facilitated the discovery of many orchid genomes, laying a solid foundation for the in-depth analysis of Dof gene families in orchids. Therefore, it is significant that we study the Dof gene family in *Cymbidium* species.

This study systematically identified 27 Dof genes in *C. goeringii*, 34 in *C. ensifolium*, and 30 in *C. sinense*. We utilized bioinformatics methods to analyze the Dof gene family in three *Cymbidium* species. In addition, we also studied the response of *CgDof* genes to heat stress and provided a set of candidate genes for heat stress resistance. This is of great significance in identifying the key regulatory factors for stress resistance in *C. goeringii.* Our findings contribute to broadening our understanding of the evolutionary relationships and functional attributes of the orchid Dof gene family, providing some new insights for further investigating the role of Dof proteins and stress breeding.

## 2. Results

### 2.1. Identification and Protein Features of Dofs

A total of 91 Dof genes were identified in three *Cymbidium* species—27 in *C. goeringii*, 34 in *C. ensifolium*, and 30 in *C. sinense*. The genes were named *CgDof1–27*, *CeDof1–34*, and *CsDof1–30*, respectively, based on their distribution order (from top to bottom) on chromosomes (Table S1). The lengths of amino acids ranged from 112 aa to 634 aa, with an average of 267.54 aa. The molecular weight ranged from 12.39 to 70.48 kDa, with a mean of 29.17 kDa. A protein is considered alkaline if its isoelectric point exceeds 7, whereas a value below 7 indicates acidic properties [25]. Only around 15.38% (14/91) of the Dof proteins have low isoelectric points (pI ≤ 7), while the average pI is 8.43. Out of 91 Dof proteins, only five (*CgDof19*, *CeDof13*, *CeDof28*, and *CsDof22*) have an average instability index below 40, indicating stability in the majority, while the average instability index (II) stands at 56.89, with 44 Dof proteins falling below this index [26]. The average aliphatic index (AI) for 91 Dof proteins ranged from 41.29 to 83.09, with an average of 56.93. Moreover, the calculated mean hydrophilic index (GRAVY) of Dof proteins in the three orchids is negative, indicating a high degree of hydrophilicity. Furthermore, subcellular localization predictions indicate that the Dof proteins of the three orchids are primarily situated in the nucleus, suggesting a potential similarity with numerous transcription factors. Subcellular localization was predicted by WoLF PSORT [27]. The protein characteristics and sequences of Dofs in the three *Cymbidium* species are shown in Table S1.

### 2.2. Phylogeny and Classification of Dofs

**Figure 1:**
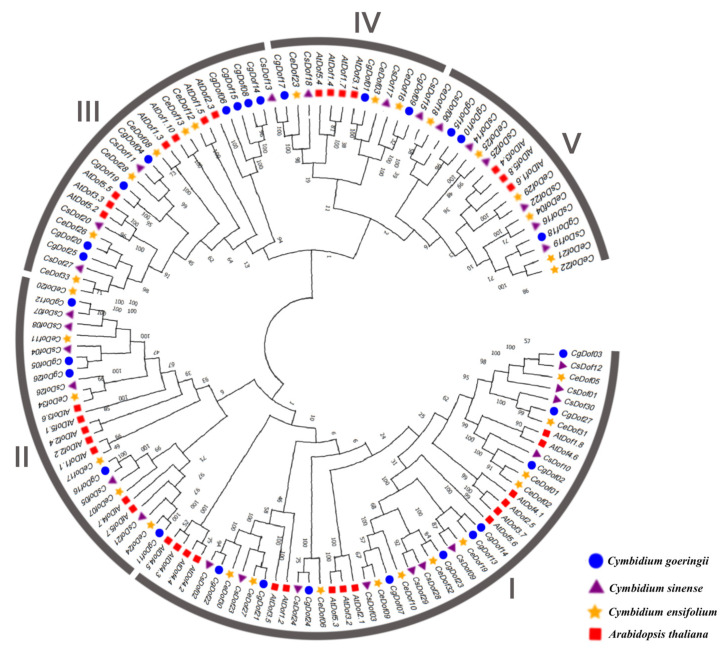
Phylogenetic tree of 127 Dof genes found in *C. goeringii*, *C. ensifolium*, *C. sinense*, and *A. thaliana*. The phylogenetic relationship of aligned sequences was estimated using the neighbor-joining (NJ) method by MEGA 11 software, and the phylogeny test was performed using 1000 replications of the bootstrap method.

The phylogenetic tree was constructed with 127 Dof genes from *C. goeringii* (27), *C. ensifolium* (34), *C. sinense* (30), and *A. thaliana* (36) (Figure 1). The phylogenetic tree showed that *Dofs* belonged to five categories, groups I–V, which is similar to the findings of previous studies [16]. We divided 127 Dof genes from four species into five groups: group I (47 genes), group II (24 genes), group III (25 genes), group IV (13 genes), and group V (18 genes). The results clearly showed that group I genes have a substantially higher number of genes than group IV genes.

### 2.3. Structure and Motif Analysis of Dofs

**Figure 2:**
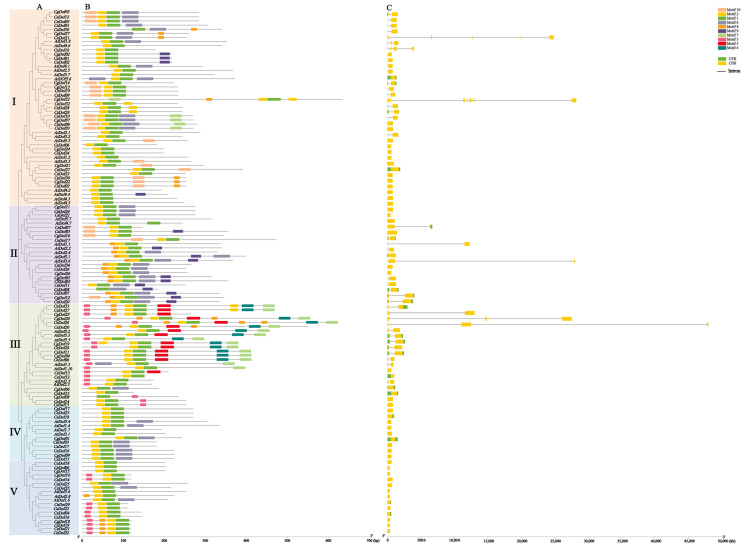
Motif and gene structure analysis of the Dof gene family in three *Cymbidium* species and *A. thaliana*. (**A**) Phylogenetic tree of 127 Dof genes; (**B**) The conserved motif of Dof proteins; (**C**) The structure of Dof proteins. TBtools v1.120 was used to integrate phylogenetic trees, conserved protein motifs, and comparative maps of gene structures.

**Figure 3:**
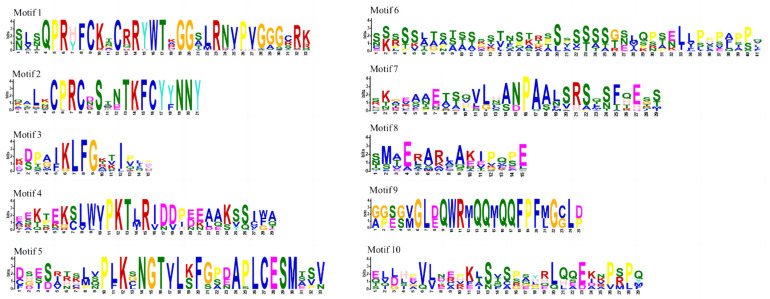
The sequence information of motifs 1 to 10. MEME Suite 5.5.5 online software was employed to analyze conserved sequence patterns of Dof genes.

Conserved motifs of 127 Dof proteins in three *Cymbidium* species and *A. thaliana* were performed through the MEME Suite 5.5.5 online website (Figure 2B). Dof proteins within clades share similar motifs, while those from different clades differ. Ten motifs were discovered in Dof proteins. Motif 1 and motif 2 are present in all Dof proteins, which may be active regions for the exercise of functions; motif 4 and motif 5 only appeared in clade III; motif 3 is found in clades III and V. The roles of Dof proteins can be attributed to the specific distribution of different structures. We observed the number and distribution of Dof protein intron-exons to further reveal the gene structure in three *Cymbidium* species and *A. thaliana* (Figure 2C). The study showed that 49.45% of the genes lacked introns, and only 50.55% of the genes contained introns, of which 8.79% of the genes had two or more introns. Among 127 Dof proteins, the number of introns ranges from zero to ten and the number of exons ranges from one to eleven. The sequence information of motifs 1 to 10 are shown individually in Figure 3.

### 2.4. Chromosomal Localization and Collinearity of Dof Genes

**Figure 4:**
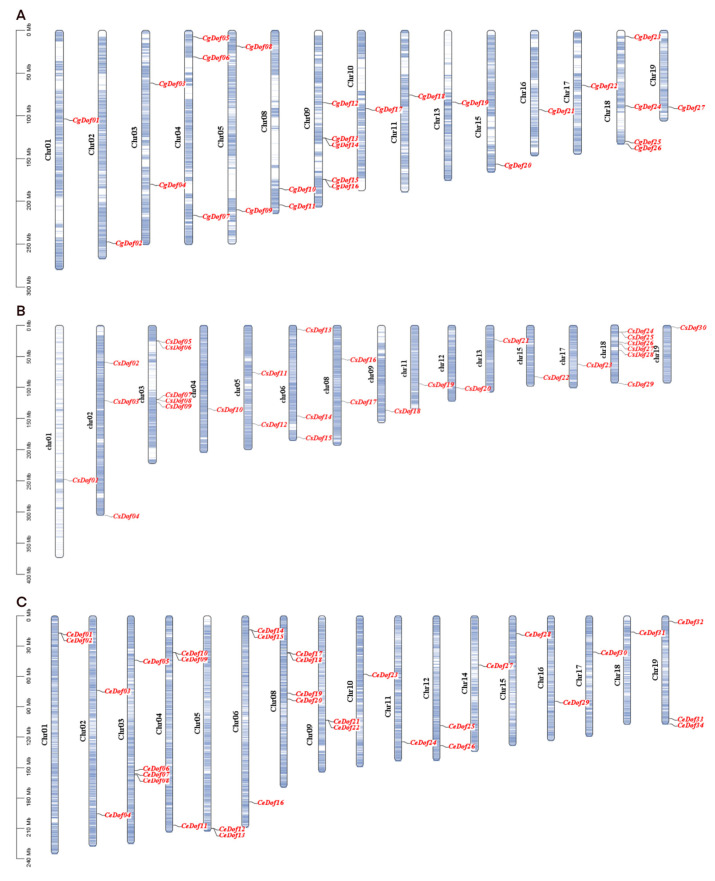
Distribution of Dofs on the chromosomes of three *Cymbidium* species. (**A**) *C. goeringii*, (**B**) *C. sinense*, and (**C**) *C. ensifolium*. The black designates the names of chromosomes, the red is employed to indicate the names of Dofs.

After analysis of chromosome location, we found that 27 *CgDof* genes were distributed on 15 chromosomes, 30 *CsDof* genes were distributed on 15 chromosomes, and 34 *CeDof* genes were distributed on 17 chromosomes (Figure 4). In *C. goeringii*, Chr 09 has the most *CgDof* genes, totaling five genes; in *C. sinensis*, Chr 08 has the most *CsDof* genes, totaling six genes; in *C. ensifolium*, Chr 03 and Chr 08 have the most *CeDof* genes, with four genes each. In addition, we identified two, three, and six pairs of tandemly duplicated genes in the *CgDof*, *CsDof*, and *CeDof* groups, respectively. These tandemly duplicated genes are closely located on chromosomes and form clusters on phylogenetic trees, indicating that they have similar functions.

**Figure 5:**
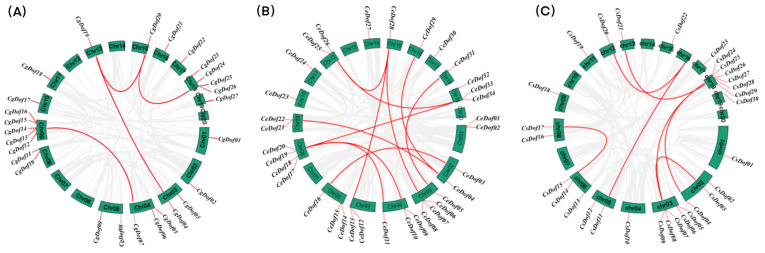
The interspecific collinearity of *C. goeringii*, *C. ensifolium*, and *C. sinense*. Red lines represent segmental duplicated gene pairs. (**A**) *C. goeringii*, (**B**) *C. ensifolium*, and (**C**) *C. sinense*.

**Figure 6:**
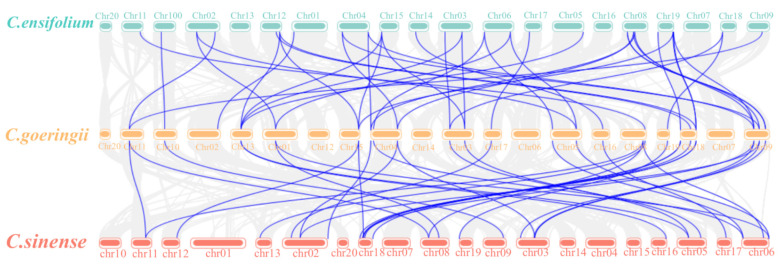
Intraspecific collinearity of three *Cymbidium* species. Blue lines depict Dof genes that exhibit collinear relationships across different species.

The intraspecific and interspecific collinearity of Dof gene sequences in three *Cymbidium* species was investigated. There are four pairs of segmentally duplicated genes in the *C. goeringii* genome (Figure 5A). The *C. ensifolium* genome had 11 pairs of segmentally duplicated genes (Figure 5B). Seven pairs of segmentally duplicated genes were present in the *C. sinense* genome (Figure 5C). There are 40 collinear gene pairs between *C. goeringii* and *C. ensifolium*, as well as 33 collinear gene pairs between *C. goeringii* and *C. sinense* (Figure 6). These results suggest that Dof genes in *C. goeringii* are more closely related to *CeDofs* than *CsDofs*.

### 2.5. Promoter Analysis of Dofs

**Figure 7:**
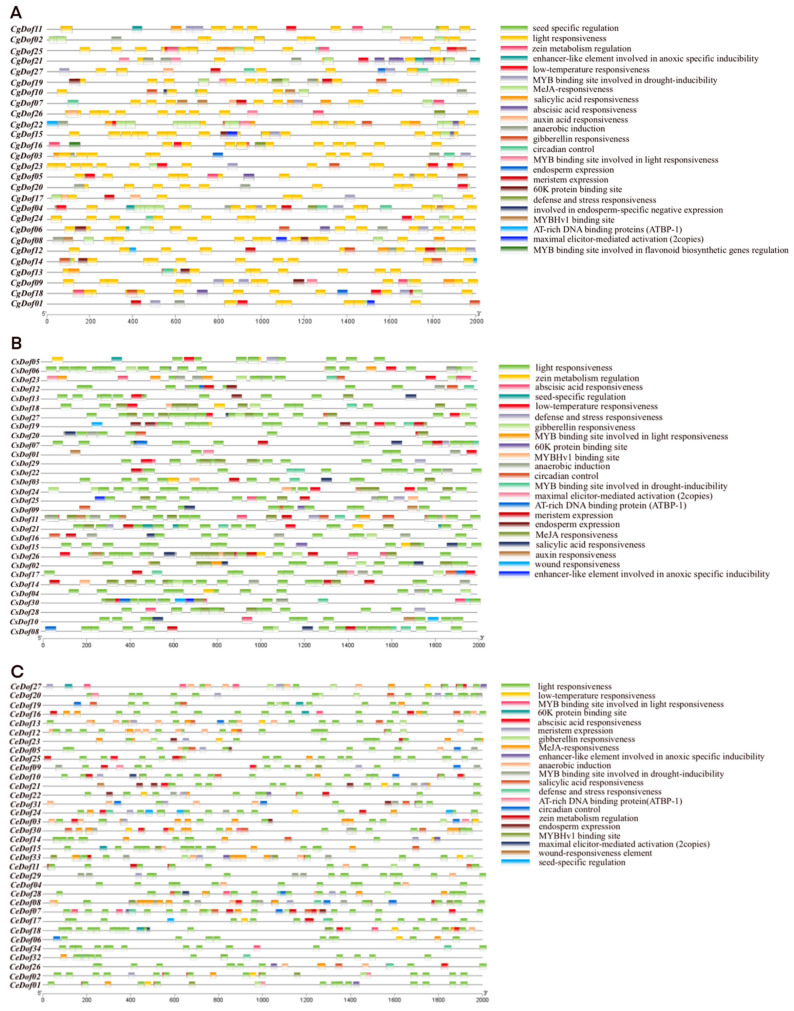
Promoter analysis of Dof genes in three *Cymbidium* species. (**A**) *C. goeringii*, (**B**) *C. ensifolium*, and (**C**) *C. sinense*.

**Figure 8:**
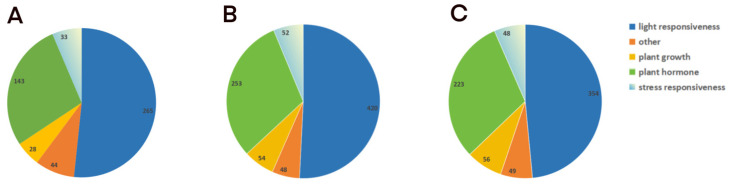
Statistics on the number of Dofs in different categories. (**A**) *C. goeringii*, (**B**) *C. ensifolium*, and (**C**) *C. sinense*. The types and numbers of Dof genes in three *Cymbidium* species are listed in Table S2.

At a distance of 2000 bp upstream of the CDS of 27 *CgDof* genes, 30 *CsDof* genes and 34 *CeDof* genes were extracted to identify *cis*-acting regulatory elements (CREs) and predict potential regulatory functions of Dof genes in three *Cymbidium* species (Figure 7). There are different types of *cis*-acting elements of the Dof gene family, which are related to plant growth and development, hormone response, light response, and abiotic stress response. The results found that the Dof genes of the three *Cymbidium* species contained more than 10 types of *cis*-acting elements, with light-responsive elements being the most prevalent, followed by hormone-responsive elements (Figure 8). In addition, some genes contain *cis*-acting elements related to abiotic stress, including low-temperature responsiveness, defense and stress responsiveness, and MYB binding sites involved in drought inducibility. A minority of genes possess *cis*-acting elements associated with plant secondary metabolism and growth development, including elements such as seed-specific regulation, zein metabolism regulation, and meristem expression. In summary, Dof genes contain various types of *cis*-acting elements, indicating that they may be involved in diverse biological processes. The types and numbers of *CgDof* genes, *CeDof* genes, and *CsDof* genes are listed in Table S2.

### 2.6. Prediction of Dof Protein Structure

An analysis of Dof proteins in three *Cymbidium* species, revealing random coils as the primary secondary structure (Table S3), offers crucial insights for further exploration into the biological functions of these proteins. Using SWISS-MODEL for homology modeling, the tertiary structures of Dof proteins from *C. goeringii* (Figure S1), *C. ensifolium* (Figure S2), and *C. sinense* (Figure S3) were predicted. Most instances of the 3D structure modeling results of Dof proteins were structurally similar in that they contained the extension chain (red part). The homology of Dof proteins in the three *Cymbidium* species and the modeling templates was almost 70%, indicating a strong structural similarity. Among SWISS-MODEL metrics, GMQE correlates positively with 3D model quality. The GMQE values of most Dof proteins were below 0.5, indicating that these proteins have high variability, while the GMQE values of relatively few proteins were above 0.5, indicating good modeling (Table S4) [28].

### 2.7. qRT-PCR Analysis of Dof Genes

**Figure 9:**
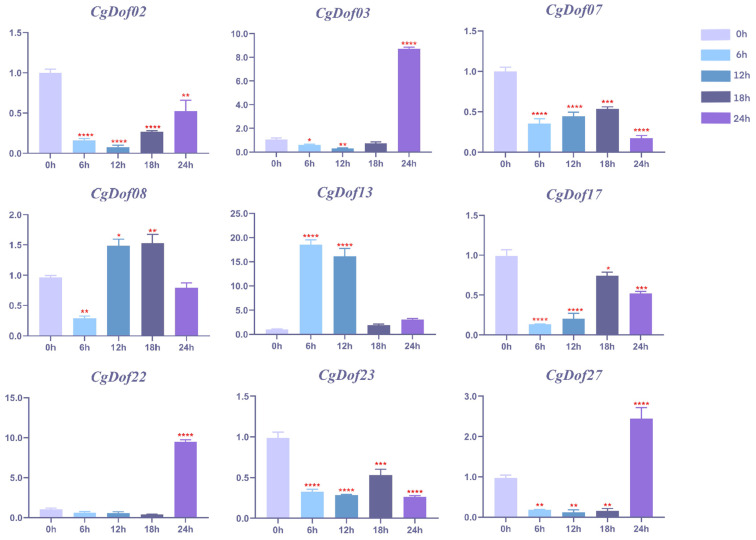
Real-time reverse transcription quantitative PCR (RT-qPCR) validation of nine *CgDofs* under high-temperature stress. The Y-axis represents the relative expression values (2^−∆∆CT^) and the X-axis represents the time of high-temperature stress. This analysis examined the status of the control sample at 0 h before high-temperature stress, and then recorded its condition after being subjected to high-temperature stress for 6, 12, 18, and 24 h. A total of three biological replicates, each with three technical repeats, were used in the experiment. Bars represent the mean values of three technical replicates ± SE. For data analysis, a student-t test was performed to identify differentially expressed genes. The red asterisk serves as an indicator denoting the significance level of the *p*-value in the respective test (* *p* < 0.05, ** *p* < 0.01, *** *p* < 0.001, **** *p* < 0.0001). Primers are shown in Table S6.

To examine the expression patterns of Dof genes under heat stress, we selected and performed RT-qPCR analyses on the nine *CgDof* genes with the highest expression levels in the leaves of *C. goeringii* (Figure 9). The results of agarose gel electrophoresis are shown in Figure S4, while the RIN (RNA integrity number) is shown in Table S7. The results of the melt curve analysis are shown in Figures S5 and S6. The FPKM values of *CgDof* genes in leaves are listed in Table S5. Notably, the expression levels of *CgDof03*, *CgDof22*, and *CgDof27* were observed to increase rapidly after 24 h under heat stress, exhibiting particularly significant upregulation. However, the expression levels of four *CgDof* genes (*CgDof02*, *CgDof47*, *CgDof17*, and *CgDof23*) were generally lower than in the control group (0 h) after heat stress. During heat treatment, the expression level of *CgDof08* increased after 12 h but then decreased after 24 h, while the expression level of *CgDof13* showed a similar pattern, increasing after 6 h and then decreasing after 18 h.

## 3. Discussion

*Cymbidium* in Orchidaceae is widely known for its unique floral morphology and floral scent characteristics [29]. Previous studies have focused on the molecular mechanisms of its growth and development, as well as complex floral development regulation processes [30,31,32]. However, there is limited research on the strategies employed by *Cymbidium* for regulating gene expression in response to abiotic stress. Dof transcription factors are a significant family of plant-specific transcription factors that play key roles in many plant biological processes, including responses to abiotic stress [13]. This study used bioinformatics analysis to investigate the evolutionary and functional characteristics of *CgDof*, *CeDof*, and *CsDof* genes, comprehensively profiling the Dof genes in three *Cymbidium* species. Furthermore, RT-qPCR experiments on nine *CgDofs* revealed variations in the expression levels of nine genes under heat stress, laying the foundation for further exploring the molecular regulation mechanism of Dof genes in plant stress resistance.

In this study, we identified 27 *CgDof* genes in *C. goeringii*, 34 *CeDof* genes in *C. ensifolium*, and 30 *CsDof* genes in *C. sinense*. The number of Dof genes in three *Cymbidium* species was higher than in *Passiflora edulis* (13) [22]; *D. huoshanense* (22) [17]; *Prunus avium* cv. ‘Tieton’ (23) [33]; *S. undatus* (26) [18]; *Cerasus serrulata* (27) [33]. However, it is relatively lower in other plants, such as *S. tuberosum* (36) [19]; *Medicago polymorpha* (36) [34]; *A. thaliana* (36) [14]; *Populus simonii* (41) [16]; *Akebia trifoliata* via (41) [35]; *Helianthus annuus* (50) [36]; *Cerasus × yedoensis* (53) [33]; *Prunus cerasus* (88) [33]. The results suggest that the Dof genes of these three *Cymbidium* species were not overextended during evolution [37]. Such variation in the numbers of Dof genes may stem from different gene duplication events or the impact of overall genome size in these diverse species [38].

In three *Cymbidium* species, 91 Dof genes were identified and their physicochemical properties were analyzed. Most proteins had instability index values above 40.0, except for four Dof proteins, including *CgDof19*, *CeDof13*, *CeDof28*, and *CsDof22*, each of which had instability index values below 40.0. In addition, the isoelectric point of most proteins (84.62%) is greater than 7. This indicates that most of the Dof proteins in these three orchid genera are stable alkaline proteins, which is similar to the Dof proteins of *D. huoshanense* [17,25,26]. Subcellular localization analysis revealed that most Dof proteins can be transported from the cytoplasm to the nucleus via nuclear transport signal domains, and some may play functions through modifications, enabling them to bind to target promoters for transcriptional activation or regulation [39]. The 127 *Dofs* are typically classified into groups I–V based on phylogenetic relationships with the Dof proteins in *A. thaliana*. The number of members of group I has far exceeded that of group IV in the course of evolution. Likewise, the Dof genes in *P. simonii* and *Triticum aestivum* also followed this classification [16,40].

The exploration of Dof protein motifs in three *Cymbidium* species is expected to provide valuable insights into their unique roles in developmental processes and stress adaptation [16]. All Dof proteins possess motif 1 and motif 2, indicating the strong conservatism of motifs 1 and 2 and their importance in determining the functional role of Dof proteins. Similar studies have also been conducted in *Spinacia oleracea*, where motifs 1 and 2 were found to be present in every Dof protein [41]. This finding parallels our research results, suggesting that motifs 1 and 2 may be key motifs within the Dof gene family, playing crucial roles in the functionality. Furthermore, the discovery that motifs 4 and 5 only exist in group III indicates the unique evolutionary significance of these motifs in this group. Gene structure analysis of Dof genes will provide valuable insights into its distinct functional role in the evolutionary process [42,43]. The majority of Dof genes belonging to the same group exhibit similar intron-exon structures. In this study, the number of introns in Dof genes ranged from zero to ten, with most members having no introns, a result similar to that of many other plant species, including *Solanum lycopersicum*, *P. trichocarpa*, *Cucumis sativus*, *Cajanus cajan*, and *Passiflora edulis* [22,44,45,46,47]. Research has found that plant genes with fewer introns exhibit stronger adaptability to the external environment and often respond quickly to stress [48]. Therefore, Dof genes in three *Cymbidium* species may be able to respond quickly to environmental changes, and these intron-free genes are the main driving force for plant tissue-specific evolution.

Gene duplication is a major factor in plant novelty and diversity and gene family expansion [49,50,51]. There are differences in the quantity and distribution of Dof genes among the three *Cymbidium* species. There are two, three, and six pairs of tandemly duplicated genes in *CgDof*, *CsDof*, and *CeDof* genes, respectively. Tandem duplications are known as a source of genetic novelty and can contribute to new genes with novel functions [52]. The collinearity analysis showed that *C. goeringii* and *C. ensifolium* had four and eleven pairs of segmentally duplicated genes, respectively, while *C. sinense* had seven. In addition, *C. goeringii* and *C. ensifolium* share 40 collinear gene pairs, and *C. goeringii* and *C. sinense* share 33 gene pairs. This suggests that *C. goeringii* is more closely related to *C. ensifolium* than *C. senense*.

*Cis*-elements play a vital part in the life cycle of plants [41]. Most *cis*-elements in the Dof gene family were associated with responses to stress, light, and hormones (Table S2). Among them, the light element is the most numerous, which is consistent with the Dof gene family of *Passiflora edulis*, indicating that the growth of these plants requires a high level of light [22]. In our study, the number of hormone-related elements in the *Dofs* ranked second. Phytohormones play a critical role in helping plants adapt to adverse environmental conditions. The elaborate hormone signaling networks and their ability to crosstalk make them ideal candidates for mediating defense responses [53]. Therefore, it is speculated that Dof genes may impact the stress resistance of *Cymbidium* species.

Previous research has shown that Dof genes can respond to various abiotic stresses. For example, the expression level of *AtDof1.1* is upregulated 2–3 times after the induction of Me-JA and *AtDof5.8* in response to salt stress [54,55]. Ma et al. found that the expression levels of *BraDof023*, *BraDof045*, and *BraDof074* were all upregulated under drought and salt stress. Low temperatures may induce the expression of *BraDof003*, *BraDof023*, *BraDof045*, and *BraDof053*, and inhibit the expression of *BraDof072* [13]. In addition, Corrales et al. found that high salt, drought, and ABA can induce CDF3 gene expression [56]. However, there is limited research into the role of the Dof gene family in heat stress. This study showed that under heat stress, the expression levels of *CgDof02*, *CgDof07*, *CgDof17*, and *CgDof23* were significantly downregulated, while the expression levels of most *CgDof* genes were markedly upregulated, indicating that the *CgDof* genes are sensitive to high-temperature stress. High temperatures can inhibit the expression of some genes and also induce the expression of *CgDof03*, *CgDof22*, *CgDof27*, *CgDof08*, and *CgDof23*. These five genes showed varying degrees of upregulation, with *CgDof03*, *CgDof22*, and *CgDof27* reaching their highest expression levels after 24 h of high-temperature treatment. The expression level of *CgDof13* increased significantly shortly after high-temperature stress, while *CgDof08* only increased significantly in the middle stage of high-temperature stress. Remarkably, most upregulated genes under high-temperature stress belong to group I, indicating that *CgDof* genes in group I have great potential for high-temperature resistance. In conclusion, our study provides new clues for the further exploration of regulatory mechanisms governing the response of orchids to heat stress.

## 4. Materials and Methods

### 4.1. Data Source and Plant Materials

The full-genome data of *C. goeringii* (NCBI: PRJNA749652) [57], *C. sinense* (NCBI: PRJNA743748) [58], and *C. ensifolium* (NCBI: PRJCA005355) [29] were obtained from the National Center for Biotechnology Information database (NCBI, https://www.ncbi.nlm.nih.gov/, accessed on 18 February 2024). Additionally, we downloaded the protein sequence of the Dofs of *A. thaliana* from The Arabidopsis Information Resource (TAIR, http://arabidopsis.org, accessed on 18 February 2024) [59]. Furthermore, the *C. goeringii* obtained in this study were cultivated in the “Forest Orchid Garden” at Fujian Agricultural and Forestry University (26°05′ N, 119°13′ E) under the shade of trees with natural light and temperature. In the cultivation environment of *C. goeringii*, the shade in summer was controlled at about 70% to simulate the light conditions of its natural growth. Three pots of mature *C. goeringii* were selected under natural growth conditions and subjected to heat stress treatments in an artificial climate culture box. Under the photoperiod of 16 h of light/8 h of darkness and 30 °C/38 °C, samples were taken at 0 h, 6 h, 12 h, 18 h, and 24 h, respectively. Subsequently, the collected samples were quickly frozen in liquid nitrogen and stored in a freezer room at −80 °C for later use.

### 4.2. Identification and Physicochemical Properties of Dof Genes

After retrieving the complete genomic sequence files of *C. goeringii*, *C. ensifolium*, and *C. sinense* from the NCBI database (https://blast.ncbi.nlm.nih.gov/Blast.cgi, accessed on 18 February 2024) [60], the protein and CDS sequence files were extracted utilizing the TBtools v1.120 software. Subsequently, 47 AtDof protein sequences were procured from the plantTFDB database [61]. Furthermore, the HMM (Hidden Markov Model) profile specific to the Dof domain (PF02701) was extracted from the Pfam database [62]. Utilizing the *AtDofs* as queries, Basic Local Alignment Search Tool (BLAST) analysis was conducted to identify putative Dof proteins in three *Cymbidium* species. The physicochemical properties of the identified Dof proteins, including amino acids, molecular weight, theoretical isoelectric points, instability index, aliphatic indexes, and Grand average of hydropathicity (GRAVY), were calculated using the ProtParam tool from ExPasy3.0 (https://www.expasy.org/, accessed on 20 February 2024) [63]. Subcellular localization prediction of proteins was performed using the WOLF PSORT website (https://wolfpsort.hgc.jp/, accessed on 20 February 2024) [27].

### 4.3. Phylogenetic Analysis of Dofs

The protein sequences of Dofs from *C. goeringii* (27 *CgDofs*), *C. ensifolium* (34 *CeDofs*), *C. sinense* (30 *CsDofs*), and *A. thaliana* (36 *AtDofs*) were merged and imported into the MEGA 7.0 software for analysis. The phylogenetic relationship of the aligned sequences was estimated using the neighbor-joining (NJ) method by MEGA 11 software. The bootstrap method was executed with 500 replicates, setting partial deletion to 50%. Then, the Phylogeny test was performed using 1000 replications of the bootstrap method. The phylogenetic tree was visualized using Evolview (http://www.evolgenius.info/evolview/#/treeview, accessed on 3 March 2024) [64].

### 4.4. Gene Structures and Conserved Motif Analysis of Dofs

Using NCBI’s CDD tool, we analyzed the conserved domains of Dof genes. In addition, MEME Suite 5.5.5 online software (http://meme-suite.org/, accessed on 3 March 2024) was employed to analyze the conserved sequence patterns of Dof genes in three *Cymbidium* species and *A. thaliana*, with a predicted number of ten [63]. TBtools v1.120 was employed to integrate phylogenetic trees, conserved protein motifs, and comparative maps of gene structures in this study [65].

### 4.5. Collinearity and Chromosomal Localization of Dofs

Multiple Synteny plot and Advanced Circos tools from TBtools v1.120 was utilized to examine the interspecific collinear relationships between *C. goeringii*, *C. ensifolium*, and *C. sinense*. Additionally, the intraspecific collinearity of these three orchid species was analyzed using the One Step MCScanX-Super Fast tool.

To analyze the distribution of Dof genes on chromosomes, the TBtools v1.120 software was employed. For collinearity analysis among the chromosomes of the three *Cymbidium* species, the One Step MCScanx command within TBtools v1.120 was utilized. Additionally, the Advance Circos package program in TBtools v1.120 was applied for displaying segmental duplications among the Dof genes.

### 4.6. Cis-Element Analysis of Dofs

To identify potential *cis*-acting elements in Dof gene promoters in three *Cymbidium* species and *A. thaliana*, we employed TBtools v1.120 software to extract 2000 bp upstream regions of these genes. Subsequently, we used PlantCARE online software (https://bioinformatics.psb.ugent.be/webtools/plantcare/html, accessed on 20 March 2024) to perform a detailed analysis of *cis*-acting regulatory elements in the extracted promoter regions of the Dof genes [66]. Data processing was carried out using Excel 2019 software for organization, and visualization was achieved with TBtools v1.120 software.

### 4.7. Protein Structure Prediction

The secondary structure of the protein is predicted by the SOPMA procedure [67]. SOPMA is a well-established method that helps us to predict the presence of various secondary structural elements, such as alpha-helices and beta-sheets, within the protein sequence. By utilizing SWISS-MODEL (https://swissmodel.expasy.org/interactive, accessed on 15 March 2024), we were able to generate a three-dimensional representation of the *Cymbidium* Dof proteins.

### 4.8. Analysis of Expression and RT-qPCR

Using a FastPure Plant Total RNA Isolation Kit (for polysaccharide- and polyphenol-rich tissues) (Vazyme Biotech Co., Ltd., Nanjing, China), RNA was isolated from the leaves of *C. goeringii*. Transcriptome sequencing and library construction were completed by Bgi Genomics Co., Ltd. (Shenzhen, China). Calculations of the gene expression level of each sample were performed using the software RSEMv1.2.8 to obtain the fragments per kilobase of transcript per million fragments (FPKM) values.

Then we employed Hifair^®^ AdvanceFast One-step RT-gDNA Digestion SuperMixfor qPCR (Yeasen Biotechnology Co., Ltd., Shanghai, China) for reverse transcription to remove the contaminated genomic DNA and generate cDNA. Hieff UNICON^®^ Universal Blue qPCR SYBR Green Master Mix was used for qRT-PCR. Primers for RT-qPCR targeting *CgDofs* were designed using Primer Premier 5 software, and their specificity was confirmed through primer blast on the NCBI website. RT-qPCR analysis was conducted using PerfectStart™ Green qPCR SuperMax (TransGen Biotech, Beijing, China). The actin gene from *C. goeringii* served as the reference gene. The expression levels of each gene were normalized to the actin internal control gene, and the relative gene expression levels were calculated by using the 2^−∆∆CT^ method [68].

## 5. Conclusions

This study examined the Dof gene family in three *Cymbidium* species. A total of 27 *CgDofs*, 34 *CeDofs*, and 30 *CsDofs* were identified for the first time, and several aspects such as the physicochemical properties, conserved motifs, exon-intron structure, and secondary/tertiary structure of proteins were analyzed. These findings indicated a high degree of conservation in Dof genes. The chromosomal localization and collinearity analysis of Dof genes in three *Cymbidium* species provided crucial information about their evolutionary relationships. The identification of hormone-responsive *cis*-acting elements in the promoter region of Dof genes helped expand the knowledge of the abiotic stress pathway in orchids. We explored the performance of Dof genes at five high-temperature stages and validated their expression patterns in leaves. Five Dof genes (*CgDof03*, *CgDof22*, *CgDof27*, *CgDof08*, and *CgDof23*) were speculated to have potential functions in the heat stress response of *C. goeringii*. These findings not only expand our understanding of the Dof gene family but also lay the foundation for a deeper understanding of their contribution to plant stress tolerance.

## Data Availability

The original data presented in the study are openly available in the National Center for Biotechnology Information (NCBI, https://www.ncbi.nlm.nih.gov/, accessed on 18 February 2024) genome database. The BioProject numbers for *C. goeringii*, *C. ensifolium*, and *C. sinense* are PRJNA749652, PRJCA005355, and PRJNA743748, respectively.

## Supplementary Materials

The following supporting information can be downloaded at: https://www.mdpi.com/article/10.3390/ijms25147662/s1.
