# Supplementary figures and images for: Genome-Based Identification of the Dof Gene Family in Three Cymbidium Species and Their Responses to Heat Stress in Cymbidium goeringii
Source: Int J Mol Sci. 2024 Jul 12;25(14):7662. doi: 10.3390/ijms25147662 (PMC11277557; doi:10.3390/ijms25147662)

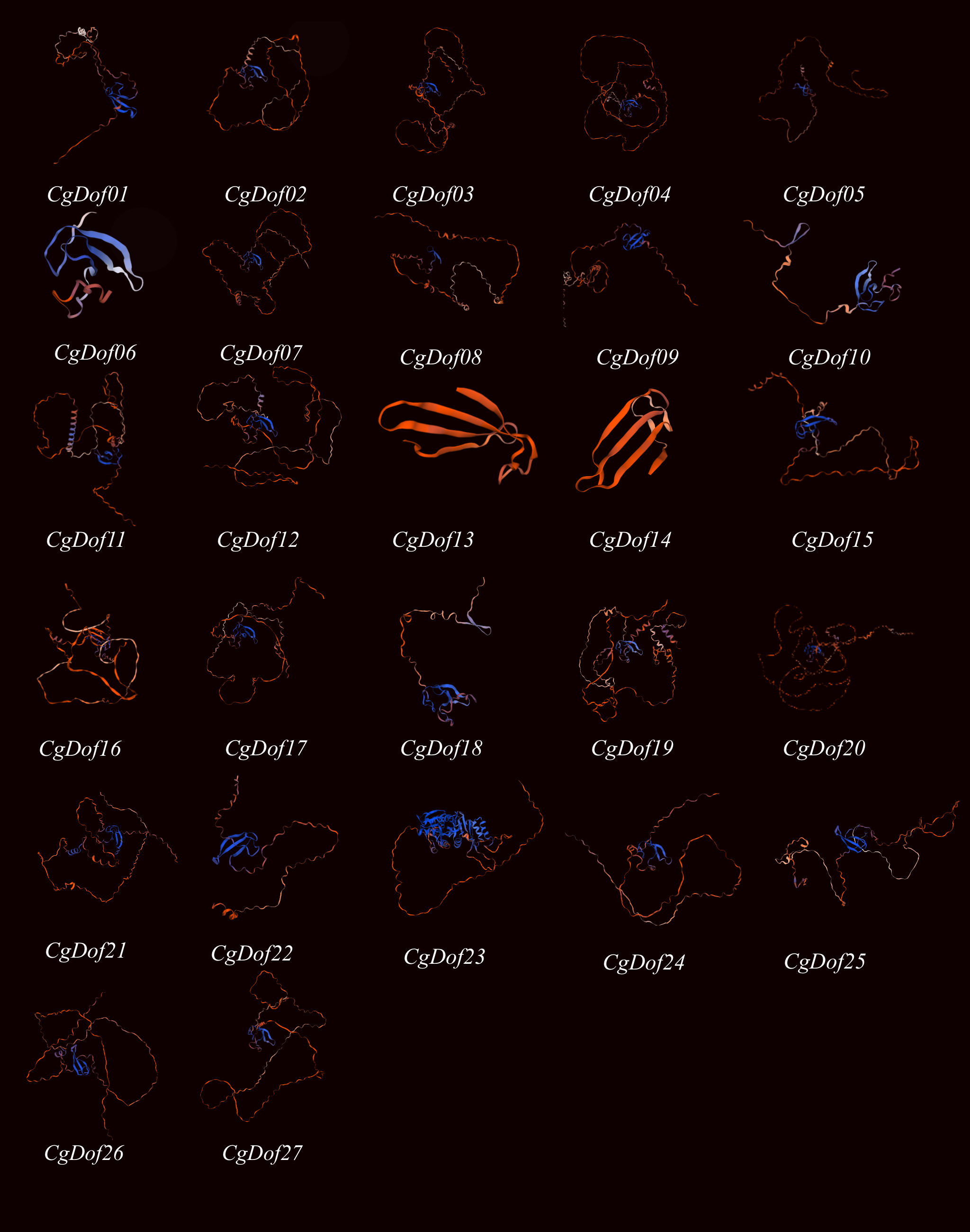

Supplement: Supplementary file 1 [file ijms-25-07662-s001.zip › Figure S1 The tertiary structures of Dof proteins from Cymbidium goeringii.png]

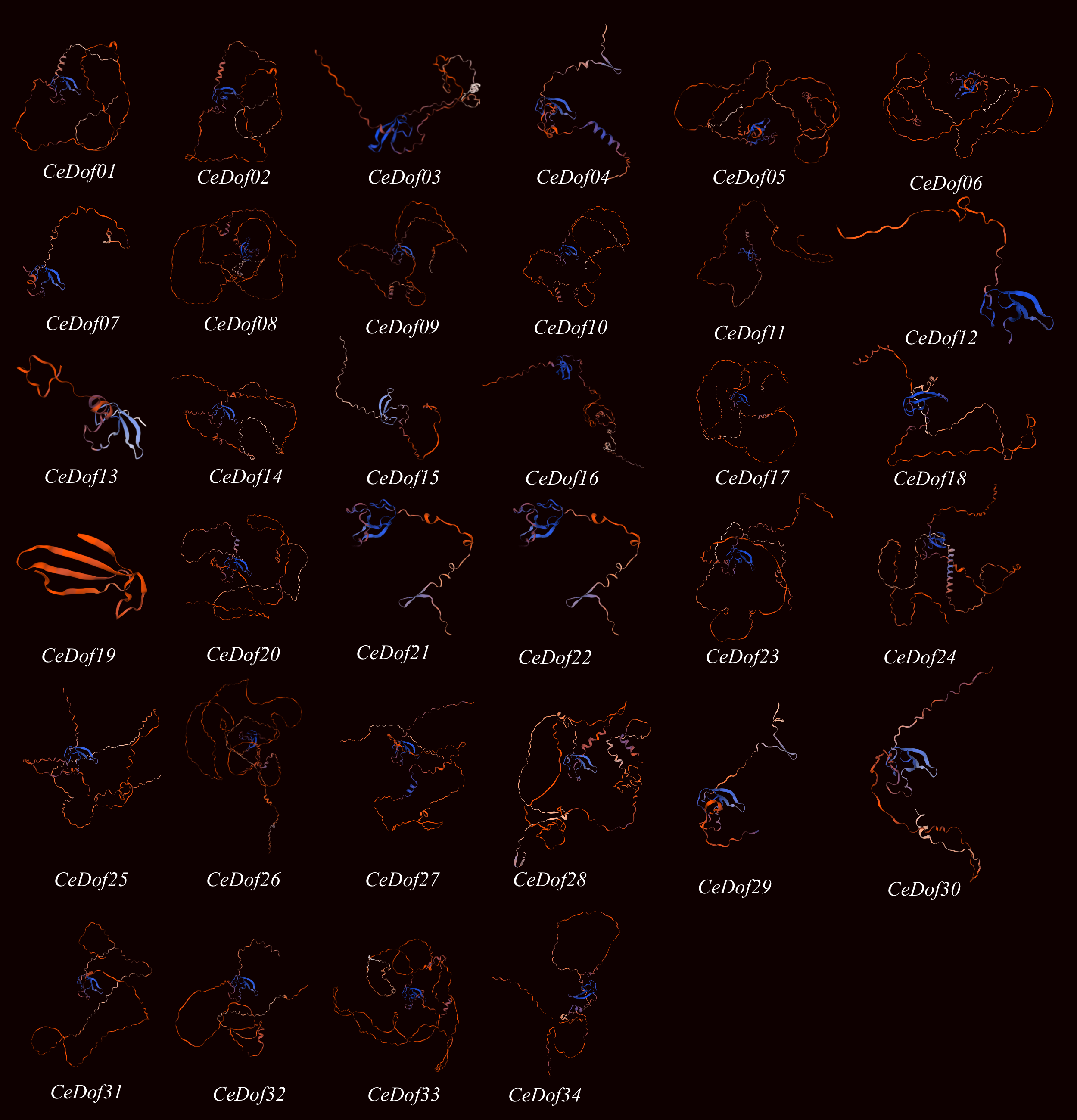

Supplement: Supplementary file 1 [file ijms-25-07662-s001.zip › Figure S2 The tertiary structures of Dof proteins from Cymbidium ensifolium .png]

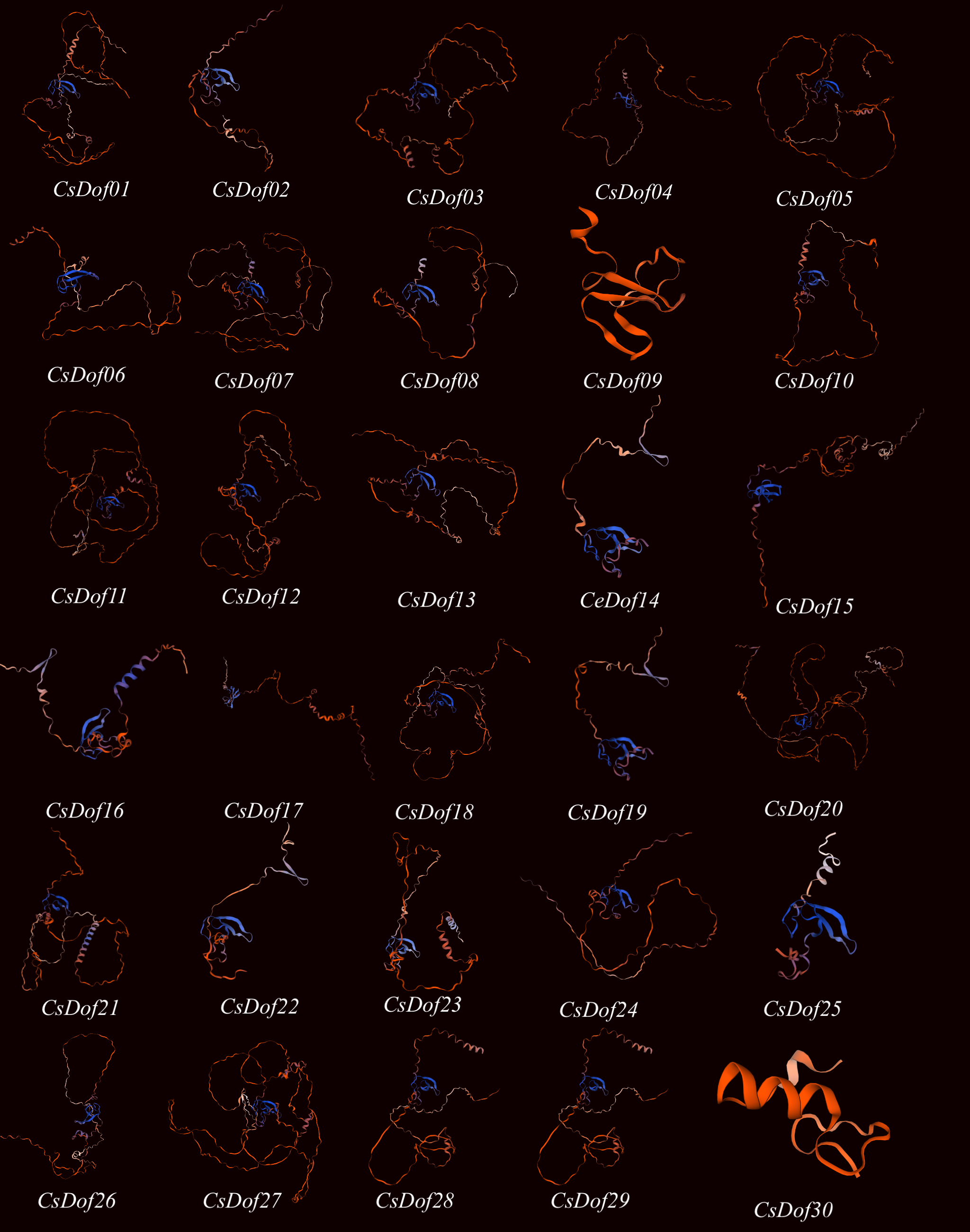

Supplement: Supplementary file 1 [file ijms-25-07662-s001.zip › Figure S3 The tertiary structures of Dof proteins from Cymbidium sinense.png]

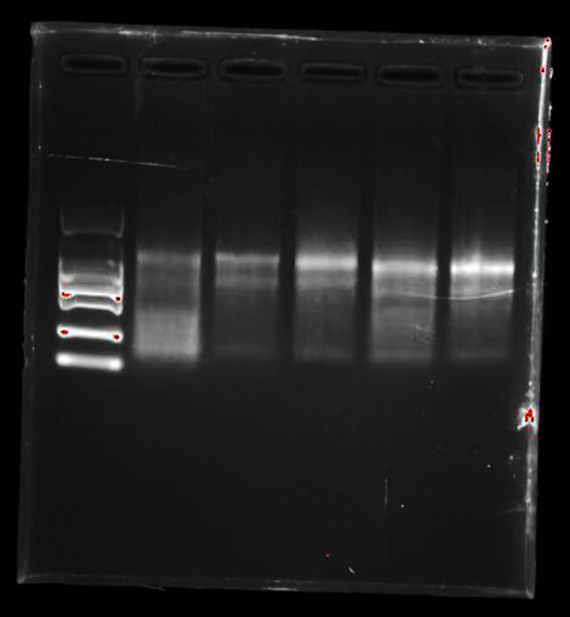

Supplement: Supplementary file 1 [file ijms-25-07662-s001.zip › Figure S4 The results of agarose gel electrophoresis.png]

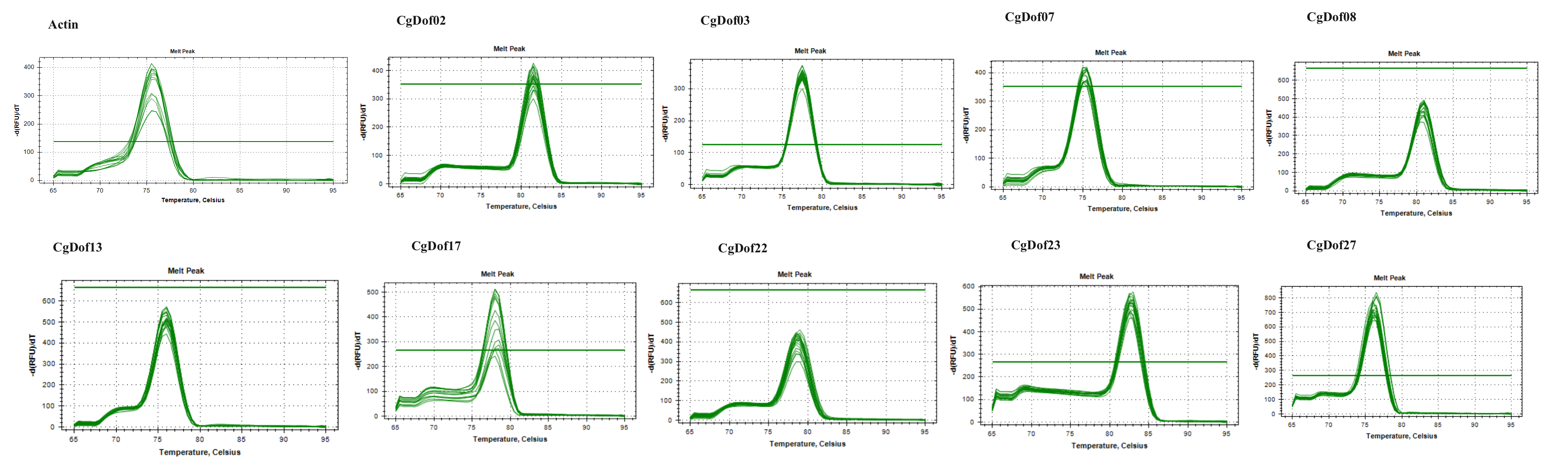

Supplement: Supplementary file 1 [file ijms-25-07662-s001.zip › Figure S5 The results of Melting Curve Analysis (Melt Peak).png]

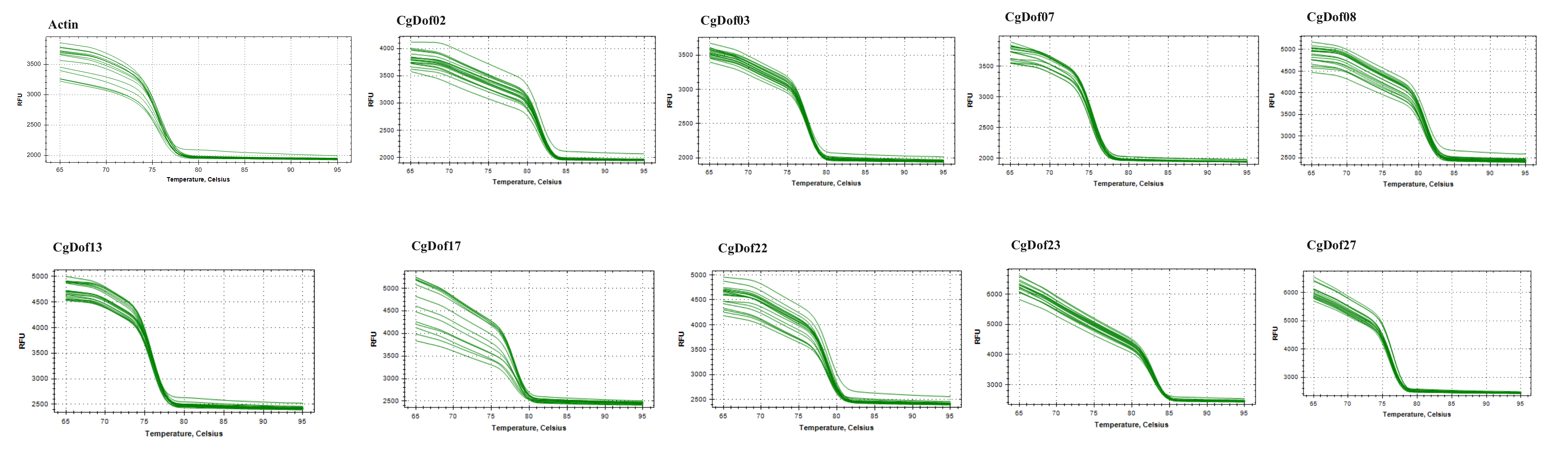

Supplement: Supplementary file 1 [file ijms-25-07662-s001.zip › Figure S6 The results of Melting Curve Analysis (Melt Curve).png]
